# Evaluating anticipatory control strategies for their capability to cope with step-down perturbations in computer simulations of human walking

**DOI:** 10.1038/s41598-022-14040-0

**Published:** 2022-06-16

**Authors:** Lucas Schreff, Daniel F. B. Haeufle, Johanna Vielemeyer, Roy Müller

**Affiliations:** 1 Department of Neurology/Department of Orthopedic Surgery, Klinikum Bayreuth GmbH, Bayreuth, Germany; 2 Bayreuth Center of Sport Science, University of Bayreuth, Bayreuth, Germany; 3 Hertie Institute for Clinical Brain Research and Center for Integrative Neuroscience, Tübingen, Germany; 4 Institute for Modelling and Simulation of Biomechanical Systems, University of Stuttgart, Stuttgart, Germany; 5 Institute of Sport Sciences, Friedrich Schiller University Jena, Jena, Germany

**Keywords:** Biomedical engineering, Computational models

## Abstract

Previous simulation studies investigated the role of reflexes and central pattern generators to explain the kinematic and dynamic adaptations in reaction to step-down perturbations. However, experiments also show preparatory adaptations in humans based on visual anticipation of a perturbation. In this study, we propose a high-level anticipatory strategy augmenting a low-level muscle-reflex control. This strategy directly changes the gain of the reflex control exclusively during the last contact prior to a drop in ground level. Our simulations show that especially the anticipatory reduction of soleus activity and the increase of hamstrings activity result in higher robustness. The best results were obtained when the change in stimulation of the soleus muscle occurred 300 ms after the heel strike of the contralateral leg. This enabled the model to descend perturbation heights up to − 0.21 m and the resulting kinematic and dynamic adaptations are similar to the experimental observations. This proves that the anticipatory strategy observed in experiments has the purpose of increasing robustness. Furthermore, this strategy outperforms other reactive strategies, e.g., pure feedback control or combined feedback and feed-forward control, with maximum perturbation heights of − 0.03 and − 0.07 m, respectively.

## Introduction

For developing hypotheses and models of motor control principles in human locomotion, researchers rely on different experimental conditions. Each experimental condition may reveal specific and important insights which can further be tested by modelling and computer simulation.

The first and fundamental experimental condition is periodic level walking. Level walking may be achieved based on central pattern generators (CPGs)^1,2^. CPGs are neuronal networks in the spinal cord that can generate rhythmic feed-forward signals^3,4^. In humans, the presence of CPGs is still discussed^5,6^. In computer simulations, CPGs allow to reproduce core characteristics of periodic walking (e.g.,^7^). In this scenario, the controller does not process any sensory information and can therefore not react to any perturbations.

A second level of experimental conditions are unexpected or camouflaged step-down perturbations (e.g.,^8–12^). Such experiments show that humans react to perturbations through reflexes. Reflex-based control is also known as feedback control and depends on proprioceptive information from receptors in the muscle–tendon complexes. Computer-based models with feedback control^13,14^ can also react to perturbations and therefore cope with them. In computer simulations, the two approaches feedback and feed-forward control are sometimes combined. In a previous study, Häufle et al.^15^ added feed-forward control to the feedback model of Geyer and Herr^13^ and showed that the interplay of different control strategies can improve the robustness of the model when descending unexpected drops in the ground.

An interesting third level of experimental conditions are visible –and therefore expected– perturbations. Such experiments reveal anticipatory adaptations, for example, in overcoming obstacles of different heights^16,17^, in the transition from steady state walking to descending stairs^18^ and step-down perturbations^19^, or in the transition from different ground stiffnesses^20^. Moreover, Müller et al.^19^ investigated the muscular adaptations that subjects make during the last ground contact prior to descending visible drops in the ground of − 0.10 and − 0.20 m. Due to the altered muscle activities, the ankle and knee joints were more flexed and the body’s center of mass (CoM) was thus lowered. This was presumably achieved by changes in muscular activities of the plantar flexors *M. gastrocnemius medialis* (GAS) and *M. soleus* (SOL). The activity of both muscles was significantly decreased depending on the perturbation height within the last 200 ms before the heel strike of the other (ipsilateral) leg occurred at the perturbed ground level height^19^. The benefit of anticipatory strategies for stability in step-down perturbations has been confirmed on the level of center of mass momentum in computer simulations^21^.

**Figure 1:**
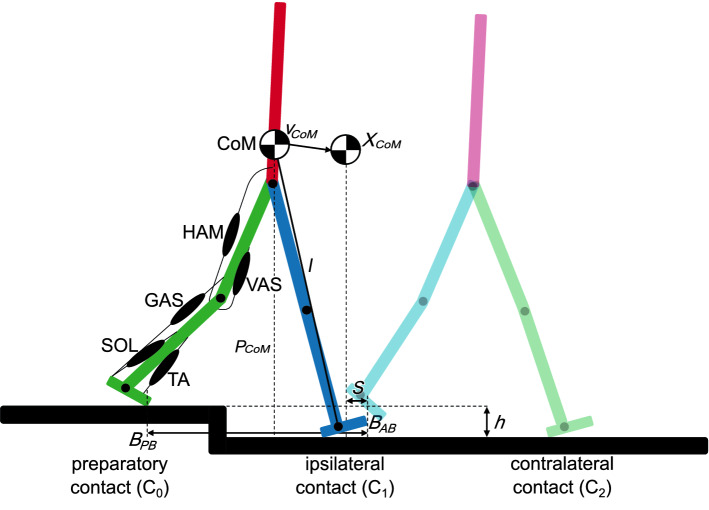
The model descends a drop in the ground with the perturbation height *h*. The contralateral leg is labelled green and the ipsilateral one blue. In the preparatory contact, the five muscles, hamstrings (HAM), vasti (VAS), gastrocnemius (GAS), soleus (SOL), and tibialis anterior (TA) are exemplarily shown in the contralateral leg. *B*_*AB*_ and *B*_*PB*_ bound the base of support in anteroposterior direction. For the calculation of the margin of stability (*S*) according to the principle of Hof et al.^22^ the horizonatal component of the CoM (*P*_*CoM*_), the distance between CoM and the ankle joint (*l*) as well as the velocity of the CoM (*v*_*CoM*_) are additionally required. *X*_*CoM*_ represents the extrapolated center of mass.

The purpose of our study is to investigate the potential benefit of anticipatory adaptations on the muscular control level. For this, we extend the walking model of Geyer and Herr^13^ to include an anticipatory control strategy. The anticipatory control strategy is employed exclusively during the last (preparatory) contact prior to a step-down perturbation (Fig. 1). In this step, we only change the synaptic gains in the reflex loops of the original model^13^ at two different times (“early anticipation”: during the entire preparatory contact C_0_, “late anticipation”: 300 ms after heel strike). The performance is evaluated in step-down perturbations. Our hypothesis is that the experimentally observed reduction in plantar flexor activity in the step prior to the perturbation^19^ will allow the model to descend larger perturbation heights. Furthermore, due to the observed interaction between perturbation height and muscle activation in all measured contralateral leg muscles (*M. vastus medialis*, *M. biceps femoris*, *M. tibialis anterior*, *M. gastrocnemius medialis*, *M. soleus*^19^) we also investigate muscle adaptations for each muscle group of the model individually (analogous to Häufle et al.^15^). In addition, we pursue the goal of finding other anticipatory strategies in which the activity of the plantar flexors is not reduced. Such strategies might be relevant in patients with spasticity in the plantar flexors or in the elderly. We compare simulation results to experimental data (CoM trajectories, ground reaction forces, leg joint kinematics, and muscle activities).

## Methods

### The basis: muscle-reflex model of human walking

We investigated the potential benefit of anticipatory strategies in computer simulations of human walking. The model used in our study is based on the muscle reflex model of Geyer and Herr^13^, which accurately predicts kinematics and dynamics of level (unperturbed) human walking. The model considers one trunk and two segmented legs consisting of thigh, shank, and foot (Fig. 1). The rigid bodies of the legs and the trunk are connected by hinge joints, actuated by 14 Hill-type muscle tendon units.

**Figure 2:**
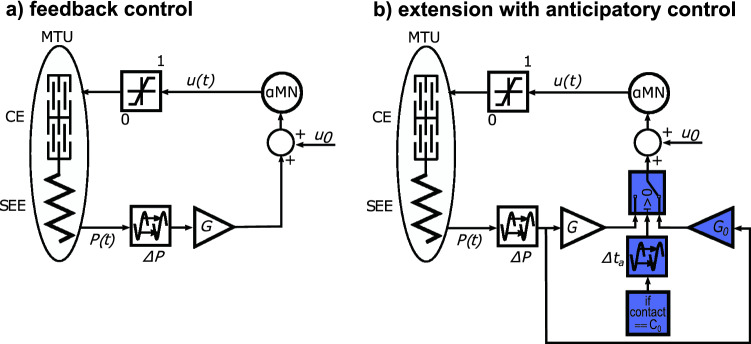
Block diagrams of (**a**) the original reflex-based walking model of Geyer and Herr^13^ and (**b**) the extension for the application of an anticipatory strategy. In both diagrams, the feedback signal *P*(*t*) from the muscle–tendon unit (MTU) is time-delayed *ΔP*, gained (*G*), and added to a constant stimulation bias (*u*_*0*_). The resulting stimulation signal (*u*_*t*_) is transmitted back to the muscle via *a*-motor neuron (*aMN*) and can only assume values in the range between 1 and 0. The extension is characterized by counting the ground contacts of the left leg. At the preparatory step C_0_, the reflex gain *G* is switched to the anticipatory gain *G*_*0*_. If *Δt*_*a*_ = 0, the anticipatory gain is active from heel strike of C_0_ (early anticipation), while late anticipation is implemented with *Δt*_*a*_ = 0.3 s. Adopted with permission from Geyer and Herr^13^

Walking is generated only by reflex-based inputs, mainly from proprioceptive muscle length and force feedbacks. Muscle stimulations of the hamstrings, hip flexors, and the gluteus additionally depend on the forward lean angle and velocity of the trunk. The feedback signals consider a neuronal time delay *ΔP* of 5–20 ms, a synaptic gain *G*, and small constant stimulation biases *u*_*0*_ (Fig. 2a). A detailed description of how the reflexes are modeled can be found in^13,23^.

### The novel extension: anticipatory control for step-down perturbations

Previous experiments showed a change in muscle activity already in the last stance phase prior to a visible step-down perturbation^19^. We term this last stance phase the “preparatory contact” (C_0_ in Fig. 1). Müller et al.^19^ found that the anticipatory strategy significantly decreased plantar flexor (i.e. *M. gastrocnemius medialis* and *M. soleus*) activity during the preparatory contact. In our model extension, the anticipatory strategy is implemented by exclusively increasing or decreasing feedback gains of one muscle in the contralateral leg only during the preparatory contact (Fig. 2b). We investigated anticipatory feedback gain adaptation in the plantar flexors gastrocnemius (GAS), and soleus (SOL) and additionally in the hamstrings (HAM), vasti (VAS), and tibialis anterior (TA). For all muscles, except the hamstrings, the gain adjustments modify the level of their proprioceptive force feedback and, consequently, their stimulation. The feedback signal we want to adapt for the hamstrings depends on the forward lean angle, velocity of the trunk, and the body weight bearing on the corresponding leg.

The experimental data of Müller et al.^19^ indicate that muscular adaptations in the plantar flexors do not occur immediately after heel strike of the preparatory contact, but only in the further course of the stance phase. This observation was also made during the transition step that subjects performed in preparation for descending stairs^18^. To test whether these late adaptations have a benefit compared to immediate adaptations after heel strike, we examine two different cases, which we refer to as “late anticipation” and “early anticipation”. For the implementation of early anticipation, we performed the gain adjustment during the entire preparatory contact (from heel strike to toe off). For late anticipation, the gain adaptation was implemented from 300 ms after heel strike (approximately midstance) to toe off. Before and after the preparatory contact, the model uses default values for the reflex gains. Geyer and Herr^13^ heuristically tuned these reflex gains for level walking to generate a robust walking pattern which resembles human joint kinematics, ground reaction forces and muscle activity patterns. The parameters were not systematically optimized for any specific cost function.

### Model analysis

To investigate the capability of the model to compensate for step-down perturbations, we performed a grid search varying perturbation height *h* and anticipatory gains *G*_*0*_. The anticipatory gain *G*_*0*_ was modified for each muscle until the feedback signal became less than 0.05 or exceeded 0.95 (corresponding to nearly deactivated or almost fully activated muscle) at any point during the preparatory step. The step-down perturbation was implemented by shifting the ground contact reference height $y_{0}$ by the perturbation height *h*:

1 $$y_{0} = 0m + h$$

The simulations start ten seconds before the perturbation allowing the model to reach a steady state walking pattern in 18 regular unperturbed steps. The simulations were continued for another ten seconds. We defined that a trial was successful when the model was still walking at the end of the simulation (20 s). With this time span we ensured that the model would either fall or return to a walking pattern after the perturbation.

To evaluate the dynamic stability of the model after the obstacle, we calculated the margin of stability (*S*) in anteroposterior direction^22,24^. The margin of stability

2 $$S = B_{AB} - X_{COM}$$

is the horizontal distance between an extrapolated center of mass (*X*_*CoM*_) and the anterior boundary (*B*_*AB*_) of the base of support, which in our case is the horizontal projections of the model´s forefoot to the ground. *X*_*CoM*_ is calculated using the horizontal component of the CoM (*P*_*CoM*_), the horizontal velocity of the CoM (*v*_*CoM*_), the acceleration due to gravity (*g*), as well as the distance between the CoM and the ankle joint (*l*) (Fig. 1).

3 $$X_{CoM} = P_{CoM} + \frac{{v_{CoM} }}{{\sqrt{\frac{g}{l}} }}$$

We determined the margin of stability at heel strike of the preparatory contact (C_0_) and the two following contacts (C_1_, C_2_) after the drop in the ground. In steady state walking, the heel has a velocity of about 1.3 m/s at heel-strike which causes deformation—not sliding—in the ground contact model. In some higher perturbation steps, the heel strike velocity is higher, and the foot is pushed forward sliding on the ground. Since this artificially increases the base of support after the heel strike, we calculate the margin of stability only when the velocity of the heel falls below 1.5 m/s to avoid these artifacts.

At heel strike of the preparatory contact, the margin of stability *S*_*C*0_ is about 0.005 m. In theory, positive values indicate a stable body configuration^22^. To be able to combine *S*_*C*1_ and *S*_*C*2_ into one value, we calculate the “shift in margin of stability”. This parameter is calculated by the mean deviation between *S*_*C*1_ as well as *S*_*C*2_ and the margin of stability (S_0_) in the preparatory contact.

4 $$\Delta S = \frac{{\left| {S_{C1} - S_{C0} } \right| + \left| {S_{C2} - S_{C0} } \right|}}{2}$$

The “shift in margin of stability” would be zero if the margin of stability of the two steps after the perturbation ($$S_{C1}$$ and $$S_{C2} )$$ would be exactly the same as the margin of stability at heel strike of the preparatory contact $${S}_{C0}$$. Comparing *ΔS* for two walking patterns indicates, that the case with smaller $$\Delta S$$ shows less deviation from the level walking pattern in terms of margin of stability and may be interpreted as “more stable”.

The model was implemented in Matlab® Simulink® R2021a and the simulations were performed with the ode15s solver (max. step size of 10 ms, relative and absolute error tolerance of 10^–3^ and 10^–4^, respectively).

### Data preparation of previously performed walking experiments

To compare the simulation results to experiments, we analyzed data of previously performed and published walking experiments^19,25^. Eight subjects (two female, six male, mean ± s.d., age: 26.8 ± 5.3 years, mass: 70.0 ± 10.6 kg, height: 180.9 ± 7.6 cm) were instructed to walk (average horizontal steady state CoM velocity 1.22 m/s) along an 8 m walkway with two consecutive force plates in its center reaching the first force plate with the contralateral and the second force plate with the ipsilateral leg. The force plate at second contact was adjustable in height as was the subsequent part of the walkway. All subjects gave written informed consent. For the expected stepdown, the track was lowered by − 0.1 m from the site of the second force plate. Spherical reflective markers were placed on the tip of the fifth toe, lateral malleolus, epicondylus lateralis femoris, trochanter major, anterior superior iliac spine, acromion, epicondylus lateralis humeri and ulnar styloid processus on both sides of the body as well as on L5 and C7 process spinosus. The CoM was determined using a body segment parameters method. Additionally, the inner angles of the knee and ankle joint were calculated.

Further information concerning the participants, and the technical details of the measurement equipment (i.e. force plates, cameras) and data processing can be found in Müller et al.^19^ and partly in Vielemeyer et al.^25^ and AminiAghdam et al.^26^.

### Ethics approval and consent to participate

The investigation was approved by the ethics review board of the University of Jena (3532-08/12) and was in accordance to the Declaration of Helsinki.

## Results

### Early anticipation

**Figure 3:**
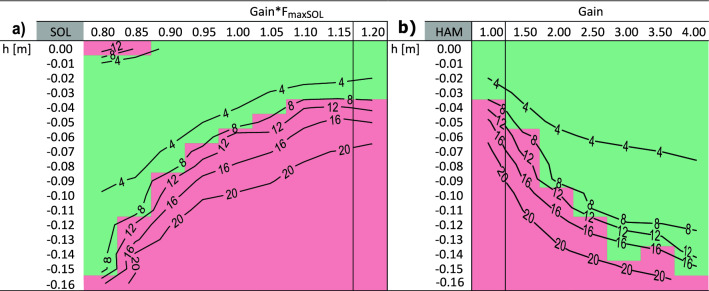
The figures (a, b) show successful (green) and unsuccessful (red) trials for various early gain adaptions and different perturbation heights *h*. Default settings of the gain, as previously described in Geyer et al.^13^, are indicated by vertical lines. For the early anticipatory SOL adaptions (**a**), the default gain setting is 1.2/*F*_*maxSOL*_ (*F*_*maxSOL*_ is the maximum isometric force of the SOL muscle). For the early anticipatory HAM adaptions (**b**) it is 1. In addition, contour lines indicate the calculated shift in margin of stability *ΔS* [cm] for each trial. For reasons of clarity, no further contour lines were drawn for *ΔS* > 20 cm.

Lowering the feedback gain of the antigravity muscles (GAS, SOL, VAS) during the entire preparatory contact (C_0_ in Fig. 1) allows the model to handle larger perturbations. Reducing the gain in SOL from 1.2/*F*_*maxSOL*_ to 0.8/*F*_*maxSO*L_ (where *F*_*maxSOL*_ is the maximum isometric force of the SOL muscle) enables the model to cope with perturbations up to − 0.15 m (Fig. 3a). For comparison: without any anticipatory adaptation the model already falls at a perturbation height *h* = − 0.04 m. For adaptations in the VAS and GAS muscles, the model tolerates drops in the ground up to − 0.07 and − 0.05 m, respectively.

The behavior of the hamstrings (HAM) is the opposite compared to the antigravity muscles. An increase of the HAM gain from 1 to 4 enables the model to descend perturbations heights of up to − 0.15 m without falling (Fig. 3b). Contrary to our expectations, anticipatory adjustments to the gain of the TA muscle bring no benefit. The TA of the model receives a feedback signal during ground contact only at the beginning and end of the stance phase. A change in the feedback gain therefore has almost no influence on the muscle activity of the TA and thus also not on the ankle angle (for more detail see supplementary material S1).

In general, the model was able to handle perturbations better if the shift in margin of stability (*ΔS*) was small (typically *ΔS* < 0.08 m). This is visible by the contour lines in Fig. 3. The margin of stability (*S*_*C*1_) at heel strike of the ipsilateral contact becomes more negative with increasing perturbation heights. This means that as a result of the perturbation the extrapolated center of mass is shifted to the front resulting in forward falling of the model. Decreasing or increasing the anticipatory gain (*G*_*0*_) of the SOL, respectively of the HAM, reduced this risk.

### Late anticipation

**Figure 4:**
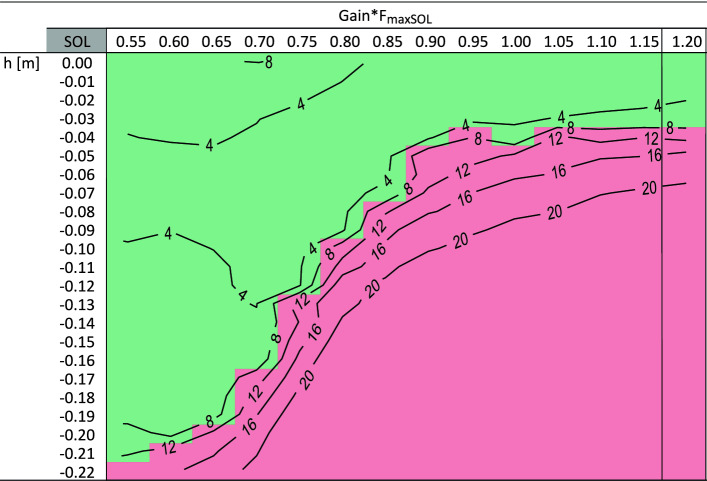
The figure shows successful (green) and unsuccessful (red) trials for various late anticipatory SOL gain adaptions and different perturbation heights *h*. Default settings of the gain, as previously described in Geyer et al.^13^, are indicated by vertical lines. Furthermore, contour lines can be seen in the figure to show in which area the calculated shift in margin of stability *ΔS* [cm] for each trial is located. For reasons of clarity, no further contour lines were drawn in the range of *ΔS* > 20 cm.

When the change in feedback gain is delayed by 300 ms after the heel strike of the preparatory contact, only the SOL shows noticeable improvements over to the early anticipations. With reduction of the feedback gain 1.2/*F*_*maxSOL*_ to 0.55/*F*_*maxSOL*_, the model can handle perturbation heights of up to − 0.21 m (Fig. 4). Supplementary Video S2 shows an animation of the trial with late anticipatory SOL adjustment (G_0SOL_ = 0.55/*F*_*maxSOL*_) at the perturbation height h = − 0.21 m.

Despite the late anticipatory gain change in GAS and HAM, simulations yield similar results to those obtained with the early anticipatory modification. The model can cope with drops in the ground up to − 0.06 and − 0.16 m, respectively. In both cases, this represents an improvement of − 0.01 m over the early anticipation. For TA and VAS, neither late gain increases nor late gain decreases bring any benefit in robustness, i.e., allow the model to descend larger perturbations than with the default gains. The margin of stability analysis revealed results comparable to the early anticipation.

### Effects on the center of mass and joint kinematics

**Figure 5:**
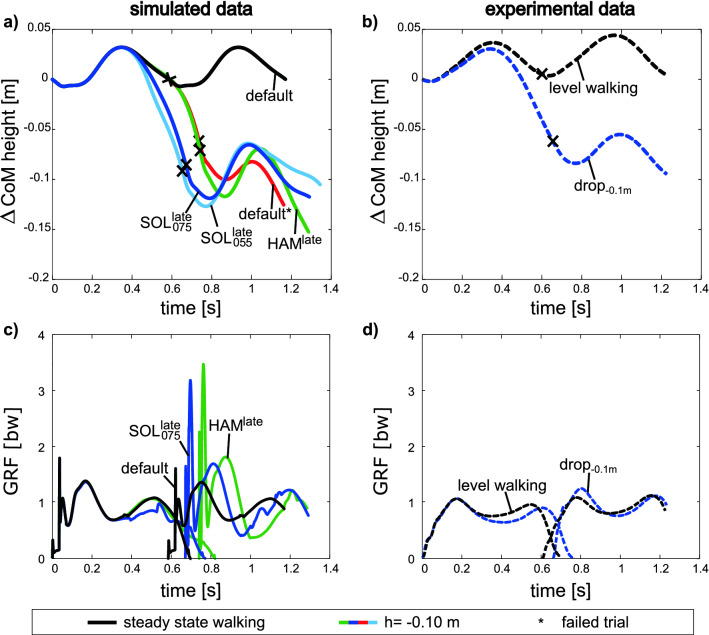
The figure shows the CoM trajectories and ground reaction forces from the heel strike of the preparatory contact (C_0_) to the heel strike of the contralateral contact (C_2_) for level and perturbed walking (perturbation height *h* = − 0.10 m). The left half of the figure presents the simulated data (**a**, **c**) (horizontal steady-state CoM velocity 1.36 m/s) and the right half presents the experimental data (**b**, **d**) (horizontal steady-state CoM velocity 1.22 m/s). The CoM trajectories are shifted by the CoM height at heel strike of the preparatory contact. For the lines of the simulated data, on one side trials with the default gain values and on the other side trials of the late anticipatory SOL (*G*_*0SOL*_ = 0.75/*F*_*maxSOL*_; *G*_*0SOL*_ = 0.55/*F*_*maxSOL*_) and HAM adjustments (*G*_*0HAM*_ = 4) were selected. For the vertical ground reaction forces, the simulated data shows a trail with default gains for level walking and trials with the SOL adjustment (*G*_*0SOL*_ = 0.75/*F*_*maxSOL*_) and the HAM adjustment (*G*_*0HAM*_ = 4) for perturbed walking. Note: The sharp peaks in the simulated data occur because in the model it is assumed that the bones are rigid bodies. In real humans, soft tissue (sometimes termed wobbling masses) comprises the majority of the weight in the legs and trunk. These can shift relative to the bones during impact and have the effect of reducing impact forces^27,28^.

Changing the feedback gain affects the CoM trajectory in the preparatory contact (Fig. 5a) and, in consequence, also the CoM position at ipsilateral heel strike (marked as “x” in Fig. 5a). The SOL strategy reduces the CoM height by about − 0.088 m (early anticipation with *G*_*0SOL*_ = 0.80/*F*_*maxSOL*_, not shown) and − 0.085 m respectively − 0.092 m (late anticipation with *G*_*0SOL*_ = 0.75/*F*_*maxSOL*_ “x” on solid blue line respectively *G*_*0SOL*_ = 0.55/*F*_*maxSOL*_, “x” on solid light blue line in Fig. 5a) at a perturbation height of − 0.10 m. The CoM is therefore lowered further than in the reference condition (“x” on red line in Fig. 5a) in which the model performs the step-down without anticipatory adaptation and therefore falls after the perturbation. In contrast, the HAM strategy reduces the CoM height by about − 0.075 m (early anticipation, not shown) and − 0.071 m (late anticipation, “x” on green line in Fig. 5a with *G*_*0HAM*_ = 4). When comparing the simulation data to the experimental data at ipsilateral heel strike, it can be seen that the CoM height for the model is at a lower height level than for the subjects (− 0.062 m, “x” on dashed blue line in Fig. 5b).

The simulation with late anticipatory $\left( {{\text{SOL}}_{075}^{{{\text{late}}}} } \right)$ adjustment (solid blue line Fig. 5c) predicts a reduced second ground reaction force (GRF) peak towards the end of the preparatory contact C_0_ by about 8% (from 1.07 to 0.98 bw) compared to the level walking (solid black line) and by about 7% (from 1.05 to 0.98 bw) compared to the HAM strategy (solid green line) trials. This matches the trend of the experimental data (second GRF peak of C_0_ decreased by 11% from 0.95 to 0.84 bw) (Fig. 5d). The ground reaction forces of the ipsilateral contact (C_1_) show that the heel strike occurs earlier for the SOL strategy than the HAM strategy (Fig. 5c). Compared to the level walking trial, both strategies predict an increased first GRF peak in the perturbed step (C_1_) by about 22% (from 1.36 to 1.74 bw) for the SOL strategy and by about 25% (from 1.36 to 1.81 bw) for the HAM strategy. However, the lower first GRF peak in the late SOL strategy is closer to the experimental data (first GRF peak of C_1_ increased by about 17% from 1.04 to 1.25 bw) (Fig. 5d).

**Figure 6:**
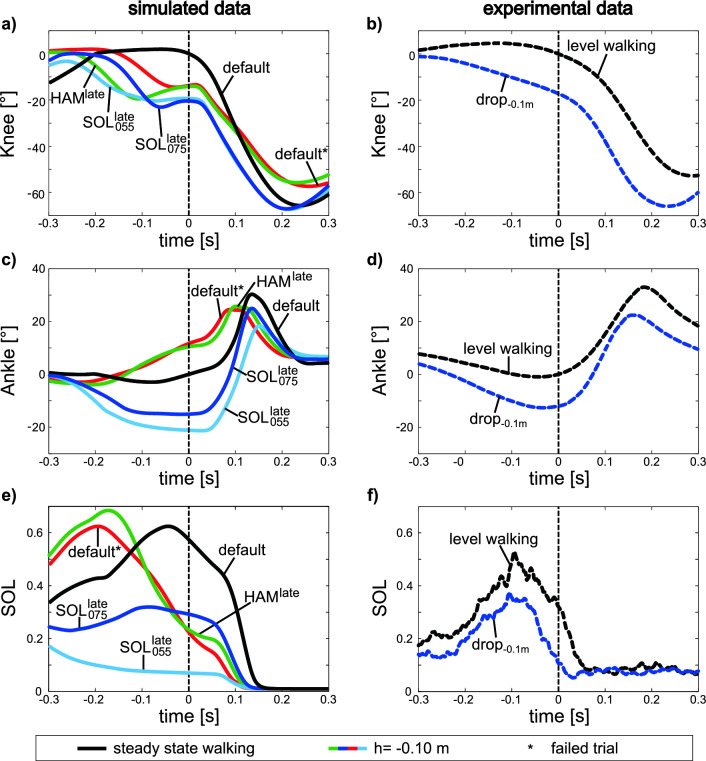
Shown are the kinematics (angle) of the knee (**a**, **b**) and ankle joint (**c**, **d**) and the muscle activity patterns (**e**, **f**) of the *M. soleus* (SOL) of the contralateral leg for level and perturbed walking (perturbation height *h* = − 0.10 m). All data are triggered to the heel strike of the ipsilateral contact (C_1_) (t = 0, vertical dashed line). For the curves of the simulated data (horizontal steady state CoM velocity 1.36 m/s), on one side trials with the default gain values and on the other side trials of the late anticipatory SOL (*G*_*0SOL*_ = 0.75/*F*_*maxSOL*_; *G*_*0SOL*_ = 0.55/*F*_*maxSOL*_) and HAM adjustments (*G*_*0HAM*_ = 4) were selected. The curves of the experimental data (horizontal steady state CoM velocity 1.22 m/s) are generated by the mean of knee and ankle joint angles from eight subjects. Note: The ankle joint angles of simulated and experimental data differ. The leg joint angles are given relative to the joint angles at the ipsilateral heel strikes of the level walking trials (solid and dashed black lines).

In the simulation, the observed adjustment in CoM height was achieved by a 6 deg more flexed knee and 27 deg more flexed ankle joint angle in the contralateral leg at ipsilateral heel strike (with respect to the reference condition, red lines Fig. 6) using the $\left( {{\text{SOL}}_{075}^{{{\text{late}}}} } \right)$ strategy (solid blue lines). Thus, the simulated data of the SOL strategy show the trends of the experimental data for both the knee joint angles (Fig. 6a, b) and the ankle joint angles (Fig. 6c, d). With the HAM strategy, the knee joint angle (solid green line in Fig. 6a) is similar to the angle occurring in the reference condition (solid red line in Fig. 6a) at ipsilateral heel strike (C_1_). Please note that the reference condition is without anticipatory adaptation and the model falls after the perturbation. The ankle angle does not show any major differences with respect to the reference line for the HAM strategy during the entire period considered. The muscle activity of the SOL is lowered during the ${\text{SOL}}_{075}^{{{\text{late}}}}$ strategy (activation peak decreased by about 48% compared to the reference trial from 0.62 to 0.32). However, in the HAM strategy, the SOL activity has a 9% higher activation peak than in the reference trial (from 0.62 to 0.68).

Supplementary Videos S3 and S4 show a comparison of the joint positions for different gain adjustments at a perturbation height h = − 0.10 m. S3 is an animation of overlaid trials with late (G_0SOL_ = 0.55/*F*_*maxSOL*_, colored) and early anticipatory SOL adjustments (G_0SOL_ = 0.80/*F*_*maxSOL*_, greyscale). The animation in S4 shows an overlay of trials with late anticipatory SOL (G_0SOL_ = 0.55/*F*_*maxSOL*_, colored) and late anticipatory HAM adjustments (G_0HAM_ = 4, greyscale).

## Discussion

Our results show that anticipatory adjustments in the preparatory contact can enhance the robustness of the model when coping with expected step-down perturbations. The maximum perturbation height that can be handled was increased sevenfold from − 0.03 m in the original reflex model of Geyer and Herr^13^ to − 0.21 m with anticipatory reflex gain adjustment. However, not all muscles equally benefit from the anticipative adjustment: anticipatory adaptations in the SOL as well as in the HAM muscle have proven to be particularly effective.

In the SOL strategy, we reduced the feedback gain of the soleus muscle and thus, lowered its activity (Fig. 6e) in the preparatory contact, resulting in curves similar to those observed in the experimental data (Fig. 6f). As a result, both the ankle and the knee joint of the contralateral leg were more flexed (Fig. 6a and c) during the preparatory contact (C_0_) and, consequently, the CoM of the model was brought to a lower height at ipsilateral heel strike (solid blue and light blue lines in Fig. 5a). These kinematic adaptations are comparable to the experimental findings (dashed blue line Fig. 5b; for more information see^19^). However, taking a closer look at the CoM height at ipsilateral heel strike it becomes obvious that the CoM was adjusted differently, i.e., about − 0.06 m in the experiments versus about − 0.09 m in the simulation. Figure 5a shows that the level of COM height reduction is dependent on the magnitude of the SOL gain adjustment. Comparing the SOL activity for the two depicted SOL adjustments *G*_*0SOL*_ = 0.75/*F*_*maxSOL*_ and *G*_*0SOL*_ = 0.55/*F*_*maxSOL*_, it can be seen that the SOL adjustment with the gain reduction of *G*_*0SOL*_ = 0.75/*F*_*maxSOL*_ better fits the experimental data. Interestingly, the model predicts that this gain reduction is just enough to allow for the − 0.10 m step-down perturbation. But it must be noted that in the simulations we only change the gain of a single muscle. In the experiments, however, targeted changes are made in all muscles. In addition, our simulations do not consider adjustments in the ipsilateral leg. For example, humans prefer toe landing (or a more plantar flexed ankle joint) for stepping down larger height differences^11,19,29^. The missing adjustments in the ipsilateral leg could also provide an explanation why the COM is lowered more in the simulations than in the experiments. Because even in the reference trial without anticipatory adaptation, the CoM (“x” on red line in Fig. 5a) is at a lower level than in the experimental data (“x” on dashed blue line in Fig. 5b). Such adjustments in the ipsilateral leg could be implemented, for example, by adding a feedforward control^15^ extending the ankle and therefore achieving earlier ground contact and less lowering of the CoM.

In our simulations, we examined early (during the entire preparatory contact C_0_) as well as late anticipatory adjustments (300 ms after heel strike). We found that the model was able to handle − 0.06 m larger perturbation heights with the late adjustments than with the early adjustments. It is noticeable that the margin of stability values S_C1_ in the successful trials of the late adjustments are less in the negative range than in those of the early adjustments at the same perturbation heights. The component in the calculation of the margin of stability that leads to this result is the horizontal CoM velocity (v_CoM_), which was lower in late adjustments. However, whether the lower horizontal CoM velocity is crucial to tolerate larger perturbation heights needs to be investigated in more detail in the future.

The literature^34–36^ suggests that in human walking it is most economical to push off pre-emptively because it reduces the subsequent collision. Also, in coping with step-down perturbations a pre-emptive push off could play a role in reducing the collision in the ipsilateral leg. In the late SOL strategy, the COM velocity is decreased right before the ipsilateral heel strike, which could point in the direction of more precise timing of contralateral push off. However, we think that the lower first GRF peak of the ipsilateral leg (Fig. 5c) in the late SOL strategy compared with the late HAM strategy, is mainly caused by the reduced COM height.

In contrast to the SOL strategy, increased gains enhance the robustness of the simulation in the HAM strategy. Thus, the HAM strategy differs from the results of the experimental investigations of Müller et al.^19^. Moreover, compared to the SOL strategy knee and ankle joints are not flexed to the same extent (Fig. 6a and c). As a result, the height of the CoM is not lowered as much, and the heel strike of the ipsilateral leg occurs at a later time (Fig. 5a). In Supplementary Video S4 it can be seen that the step length at step-down is larger for the HAM strategy than for the SOL strategy. The longer step length allows better control of the forward horizontal momenta after contact on the perturbed ground level^30^.

Although the HAM strategy is not observed in experimental investigations with young (age: 26.8 ± 5.3 years) subjects^19^, it could be used by other groups of people. Compared to young adults, older people show a higher co-contraction in the leg joints when performing step-down movements (e.g.,^31,32^). For example, when descending the first step of a staircase, the co-contraction in the ankle and knee joints of the contralateral leg is several times higher in the elderly^33^. The high co-contraction might conflict with the lowering of the SOL muscle activity and therefore speculatively favor the HAM strategy in elderly subjects. The HAM strategy could also be considered for people with spastic plantar flexor muscles. However, these speculations still need to be investigated in more detail.

It has already been shown that varying reflex gains in the walking controller of Geyer and Herr^13^ allows to generate a wide variety of movement tasks, such as overstepping obstacles and walking on inclined surfaces^14^. For the application of our HAM or SOL strategy, we changed only a single gain parameter during a single ground contact. The anticipatory adjustments therefore are built on the feedback control of the walking model and are not implemented using another control approach. Such anticipatory adaptation of a reflex gain based on processing of environmental cues could be interpreted as a high-level controller efficiently relying on the low-level reflex strategy. This is interesting from a hierarchical motor control point of view (e.g.,^37,38^), but also in terms of finding control patterns e.g., by reinforcement learning^39,40^.

The muscle reflex model of Geyer and Herr^13^, extended here to include anticipatory adaptation of muscle gains during the last contact prior to a step-down perturbation, could be further modified in the future. Experimental data in the literature indicates that anticipation can happen already in steps before C_0_ (so C_−1_, C_−2_ etc.). In the subjects of the studies^18,41,42^, a reduction of CoM velocity and step length was already observed at these steps. Similar findings could be obtained through motion-optimized simulations by Darici et al.^21^. It remains to be examined whether the adjustments made in C_−1_ and C_−2_ ensure that the reflex model can handle larger perturbation heights.

A benefit of robustness was also previously observed in the same model by combining feedback and repetitive feedforward control^15^. The focus there was on the experimental conditions of level periodic walking and reactive walking in the presence of unexpected perturbations. The combination led to a maximum perturbation height of − 0.07 m and also showed to be beneficial in other muscles groups, e.g., VAS^15^. The anticipatory strategy implemented here outperforms the reactive & repetitive strategy threefold. As all of these simulations were performed on the basis of the same reflex-based walking controller^13^, this comparison sheds light on the relative benefit of such control strategies and is therefore a first step towards a quantitative comparison of the many components of walking control^43^.

## Supplementary Information

Supplementary Information 1.

Supplementary Video 1.

Supplementary Video 2.

Supplementary Video 3.

## Data Availability

The datasets generated and analyzed during the current study are available from the corresponding author on reasonable request.
