# Supplementary material for: Evaluating anticipatory control strategies for their capability to cope with step-down perturbations in computer simulations of human walking
Source: Sci Rep. 2022 Jun 16;12:10075. doi: 10.1038/s41598-022-14040-0 (PMC9203805; doi:10.1038/s41598-022-14040-0)
Supplement: Supplementary file 1 — Supplementary Information 1. [file 41598_2022_14040_MOESM1_ESM.pdf]

Electronic supplementary material S1

Evaluating anticipatory control strategies for their capability to cope with step-down perturbations in computer simulations of human walking

In this electronic supplementary material, we describe in more detail why anticipatory gain adjustments in the tibialis anterior (TA) do not lead to increased robustness in the muscle reflex model of Geyer and Herr [1].

A change in the feedback gain has hardly any effect on the ankle angle of the model because the TA only receives a feedback signal at the beginning and end of the stance phase (see muscle activity of the TA with default gain (1.1 blue curve) in the Fig. S1a). In our investigations, we increased the feedback gain from 1.1 to 10. The figure below shows that even when the gain is increased to 100 (orange curve), the TA is not continuously highly activated during the stance phase. The resulting small change in the ankle joint angle can be seen in Fig S1b.

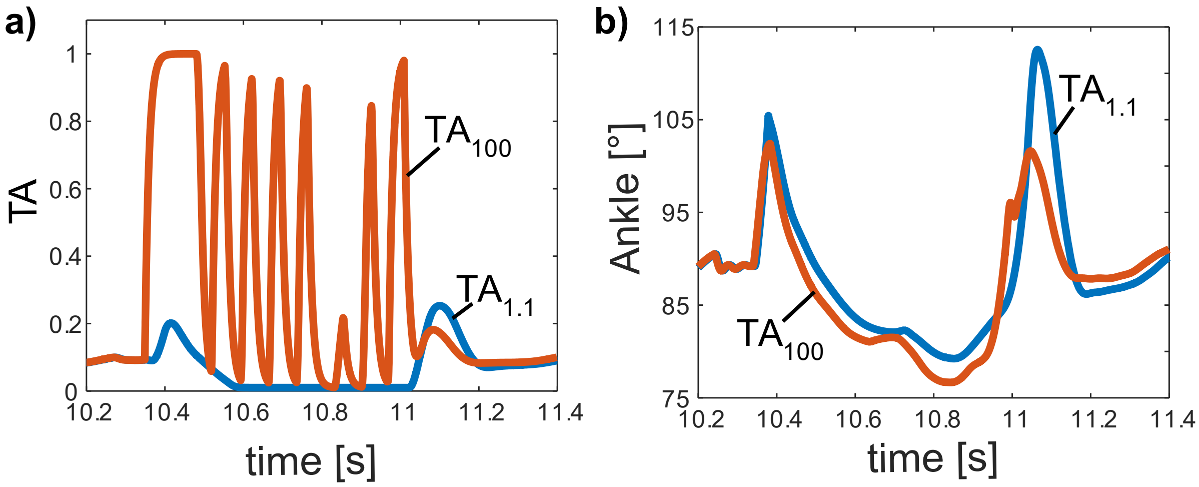

Figure S1. The figure shows the muscle activity patterns of the TA (a) and the kinematics of the ankle joint (b) during stance phase for G_{0TA}=1.1 and G_{0TA}=100.

References

1. Geyer, H. & Herr, H. A muscle-reflex model that encodes principles of legged mechanics produces human walking dynamics and muscle activities. *IEEE Trans. Neural Syst. Rehabil. Eng.* **18,** 263–273; 10.1109/TNSRE.2010.2047592 (2010).
